# Distance From the Foveal Center: A Method for the Calculation of Eccentric Fixation

**DOI:** 10.1167/tvst.14.5.9

**Published:** 2025-05-06

**Authors:** Thales A. C. de Guimaraes, Angelos Kalitzeos, James Bainbridge, Michel Michaelides

**Affiliations:** 1 UCL Institute of Ophthalmology, University College London, London, UK; 2 Moorfields Eye Hospital NHS Foundation Trust, London, UK; 3 Department of Ophthalmology, Faculdade São Leopoldo Mandic, Campinas, SP, Brazil

**Keywords:** foveal center (FC), fovea, preferred retinal locus (PRL), eccentric fixation, inherited retinal dystrophy

## Abstract

**Purpose:**

The purpose of this study was to describe a method to determine the position of the preferred retinal locus (PRL).

**Methods:**

Cross-sectional data were obtained prospectively. Microperimetry was completed with the Macular Integrity Assessment (MAIA) under mesopic testing conditions. Spectral domain optical coherence tomography (SD-OCT) was acquired with the Spectralis SD-OCT system. The printout of the MAIA containing the PRL and both the horizontal and vertical transfoveal scans were interpolated in Adobe Photoshop, which was used to calculate the foveal center (FC) and to create a custom ruler to measure the distance from the foveal center (DFC) and the distance between PRL (DPRL). The methodology was tested by two independent graders in participants of a natural history study for *KCNV2*-associated retinopathy.

**Results:**

Twenty-two eyes of 12 subjects were analyzed at a mean age of 31.9 years (range = 11–54 years, SD = ±14.3). The mean DFC and DPRL was 1398 µm (range = 182.8–2896, SD = ±755.4), and 751.4 µm (range = 144.5–1493.3, SD = ±458.3) in the right eyes, and 1104 µm (range = 341.8–2513, SD = ±653.78) and 742.5 µm (range = 120–1918, SD = ±586.5) in the left eyes, respectively. There was no significant interocular correlation of DFC (*r* = −0.036, *P* = 0.92) or DPRL (*r* = 0.41, *P* = 0.26), or between the two variables in the right (*r* = 0.519, *P* = 0.084) and left eyes (*r* = 0.014, *P* = 0.97). These suggest that DPRL may not be related to the location of eccentric fixation and that the values of either eye are independent of each other.

**Conclusions:**

Our data suggest that these are reliable parameters, which could be of relevance in the context of clinical trials. The sample size is small and its correlation with other functional and structural outcomes remains to be explored, but these findings provide a framework for further development.

**Translational Relevance:**

This work bridges the distance between basic science and clinical care by providing a reliable and replicable method to quantify the preferred retinal locus of patients with eccentric fixation.

## Introduction

Historically, regulatory agencies have adopted function instead of structure as the primary endpoint in clinical trials. The most widely used outcome is a change in best-corrected visual acuity (BCVA) of 15 or more Early Treatment of Diabetic Retinopathy Study (ETDRS) letters, although it carries several limitations. For instance, it is known to be limited in tracking mild progression, ceiling and floor effects, as well as in assessing functional deficits in early disease.^1^ Hence, the importance of psychophysical tests, particularly pertaining to retinal sensitivity, cannot be overstated.

There is a wide range of modalities to characterize individuals functionally. Among the most commonly used are static perimetry and microperimetry (also known as fundus-guided perimetry). These allow for in-depth functional characterization of individuals affected with various forms of retinal disease, particularly in the field of inherited retinal diseases. Moreover, cone and rod photoreceptor function can be isolated under different testing conditions, namely photopic and scotopic, respectively, or simultaneously (mesopic).^2^

Despite the considerable advances in the field, eccentric fixation remains a relatively unexplored metric. Indeed, to the authors’ knowledge, this parameter has yet to be considered as a robust functional outcome in clinical trials. There are several ways in which the preferred retinal locus (PRL) can be described, including the area of an ellipse (in squared degrees), and more qualitative descriptions, such as the quadrant position of the PRL. The main limitation with approaches such as the bivariate contour ellipse area (BCEA), is that it assumes a bivariate normal distribution to the position of the fixation target on the retinal imaging. However, as described previously, the pattern of retinal imaging positioning is rather task-dependent, not conforming with a normal distribution.^3^^,^^4^ Alternatively, some research groups have successfully used structural tests, such as optical coherence tomography (OCT), to assess eccentric fixation in subjects with microtropia and residual amblyopia. This was managed by manually measuring the position between the retinal fixation point and the anatomical fovea using software tools (expressed in µm),^5^ or by calculating the OCT fixation shift (in degrees), the latter being correlated with visual acuity.^6^ Several other studies have been previously described using various imaging modalities for a wide range of macular diseases.^7^^–^^10^

In the present paper, the authors describe and propose a method to obtain a uniform and repeatable measure derived from a combination of a functional (microperimetry) and a structural (OCT) modality. This methodology was applied in a cohort of patients with a form of inherited retinal dystrophy affecting predominantly central vision caused by disease-causing variants in the *KCNV2* gene (OMIM *607604).

## Materials and Methods

This study adhered to the tenets of the Declaration of Helsinki. Informed consent was obtained for all participants.

### Patient Identification

All subjects were participants of an ethically approved natural history study for *KCNV2*-associated retinal dystrophy in a tertiary referral center (Moorfields Eye Hospital, London, UK).^11^ Subjects were seen prospectively every 6 months for 2 years. The data were assessed cross-sectionally at their third visit (year 1) with the aim of reducing the influence of learning effects. The ETDRS BCVA was acquired on the same day as part of the natural history study protocol and will be described for descriptive purposes (in LogMAR).

### Macular Integrity Assessment

**Figure 1.**
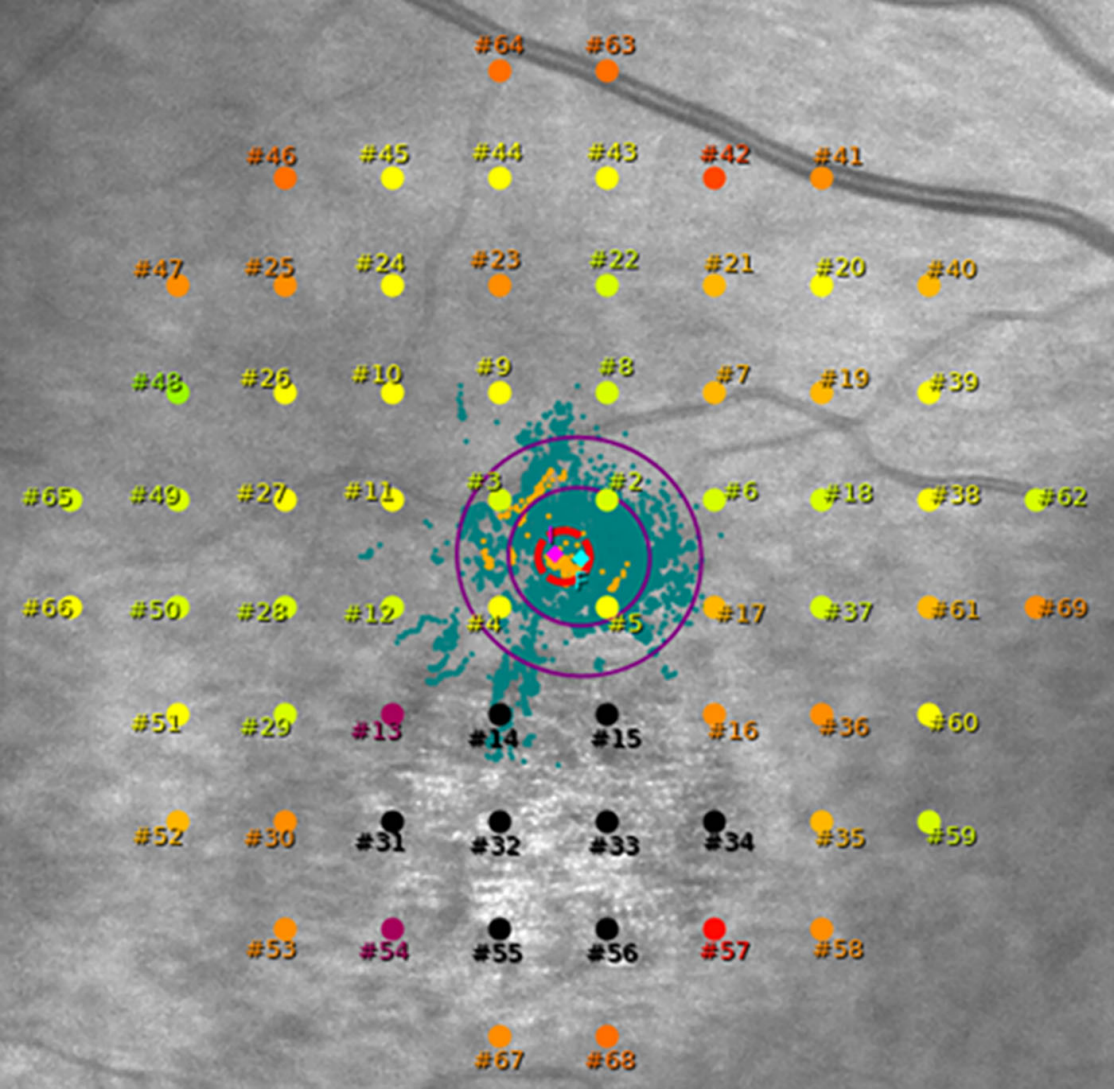
The 10–2 grid used for mesopic microperimetry. The *red circle* in the middle is the fixation target size and the two *lozenge-like shapes* (*pink* and *cyan*) are the preferred retinal locus initial (PRLi) and final (PRLf). These are surrounded by two ellipses (BCEA), encompassing the cloud of fixation points (in *green*). The patient in question is affected by *KCNV2*-associated retinal dystrophy, with the central scotoma (*black* test points) corresponding to an area of atrophy in the infrared image.

The Macular Integrity Assessment (MAIA; CenterVue, Padova, Italy) was used to assess mesopic retinal sensitivity. Figure 1 demonstrates the grid used for the patients with each of the 68 stimuli numbers.

The test was conducted monocularly in a darkened room without pharmacological mydriasis. Prior to the test, the subjects were dark adapted for 20 minutes. The contralateral eye not being tested was occluded – with the right eye being tested first in each case. A Goldmann size III stimulus with duration of 200 ms was used in a 4–2 threshold strategy and background luminance of 1.27 cd/m^2^. The fixation target was a red circle, as standardized by the MAIA device. The central 10 degrees were tested using a standard 10–2 grid consisting of 68 points with test points spaced 2 degrees apart. This device has a dynamic range of 36 decibels (dB). Due to eccentric fixation, if necessary (e.g. if the PRL was too far from the posterior pole), the grid position was manually changed to the anatomic fovea by using the spectral domain optical coherence tomography (SD-OCT) as reference. This was done to test the greatest possible number of points in the central retina. The reliability criterion chosen for the MAIA was a threshold of fixation loss ≤ 15%, with repetition of the test should it exceed this value. Catch trials are not available in the MAIA, hence this is the only reliability index available for the device.

### Preferred Retinal Locus and Fixation Stability

The PRL is automatically assessed by the MAIA, which provides accurate and objective information regarding retinal location and stability of fixation. It tracks eye movements 25 times per second for 10 seconds and plots the resulting distribution over the scanning laser ophthalmoscopy (SLO) image. Two main PRL reference points, known as PRL_initial (PRLi) and PRL_final (PRLf), are calculated at the center of the cluster of fixation points. The former defines the center of the stimuli grid (after the initial 10 seconds of examination), whereas the latter is found at the end of the examination and serves as the reference point used to calculate fixation stability. The PRLf is invariably calculated at the end of the test and is dependent on the total test time of each subject. MAIA calculates fixation stability in two different ways:

1. As the area of a 2-dimensional ellipse –BCEA – which encompasses the cloud of fixation points based on standard deviations of the vertical and horizontal eye positions during the fixation attempt, and
2. By calculating the percentage of fixation points located within a distance of 1 (P1) and 2 degrees (P2) from the reference point (PRLf), respectively: (i) if more than 75% of the fixation points are located within P1, the fixation is classified as “stable”; (ii) if less than 75% are within the P1, but more than 75% within the P2, it is classified as “relatively unstable”; and (iii) if less than 75% are located within the P2, the fixation is classified as “unstable.” Both the BCEA and the PRLf (cyan lozenge) are shown in Figure 1.

Although these two methods essentially constitute two ways of calculating a similar outcome, unlike the BCEA, the PRLi and PRLf provide a more “pinpoint” location to the fixation, particularly the PRLf, given it accurately summarizes and represents the center of the fixation in context with the retinal imaging, thus facilitating the visualization of the PRL.

### Retinal Imaging

All subjects were imaged using the Spectralis SD-OCT system (Heidelberg, Engineering Inc., Heidelberg, Germany). The test was conducted in a darkened room after pupil dilation with 1 drop of 2.5% phenylephrine and 1% tropicamide. Twenty degrees square volume scans were obtained, both in the vertical and horizontal axis (193 B-scans), with ART off. Subsequently, 20-degree wide single line scans were obtained vertically and horizontally. In the presence of significant nystagmus, the number of averaged images were reduced.

Fundus autofluorescence (FAF) was then undertaken for each eye. The autofluorescence mode has a 488 nm excitation and a beam power of less than 260 microwatts. Thirty degrees square images were obtained.

### Measuring Fixation Shift

**Figure 2.**
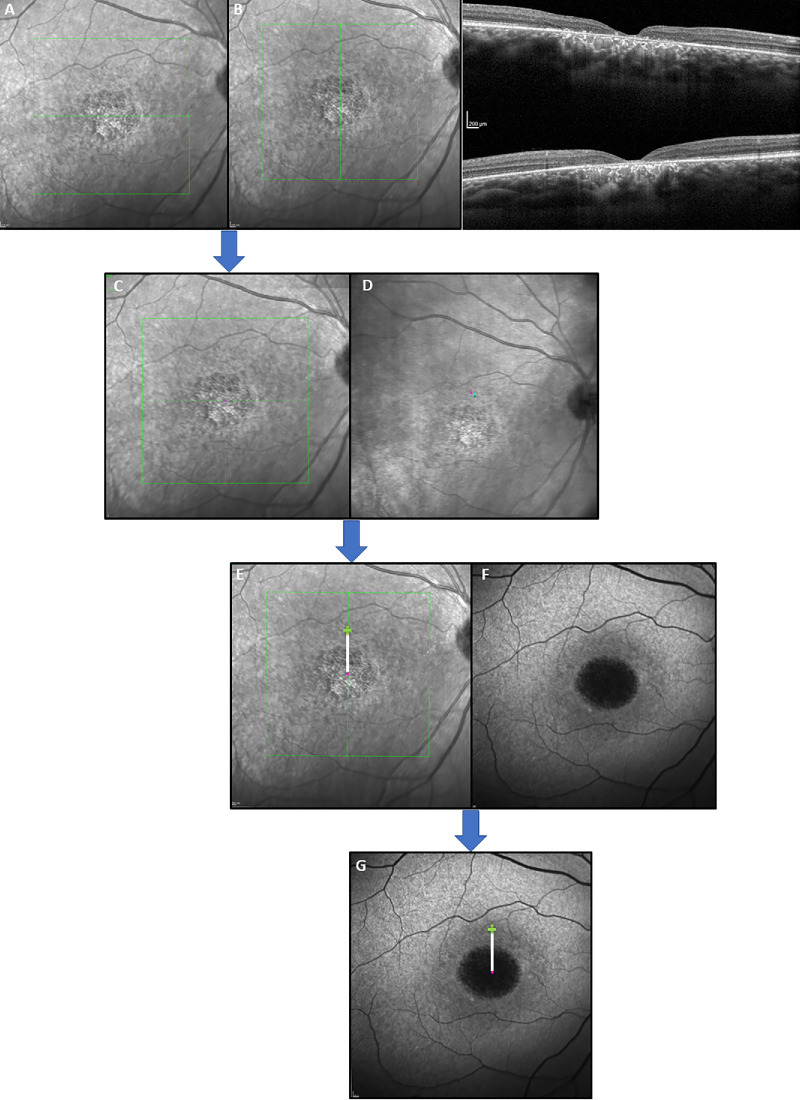
Montage showing the steps followed to obtain the distance from foveal center (DFC). (**A, B**) Shows the selected vertical and horizontal scans that represent the most anatomically accurate foveal center (FC). (**C**) The FC is obtained after overlapping the two images by matching the vessels and optic nerve silhouette, with the aid of the transparency function in Photoshop. This image is then overlapped with the MAIA infrared image (IR) shown in (**D**). Of note, this IR image is a “simplified” printout that is available directly in the device, which is highly customizable. In this case, to reduce the amount of visual clutter, the image was exported containing only with the PRL, as shown in **D**. The MAIA export allows the selection of the piece of information needed in the final printout – for the purposes of this montage, only the PRLi and PRLf were left in the image. (**E**) A *green cross* is made after overlapping **C** and **D** and the shortest straight line between FC and PRLf is traced. The resulting image is then overlapped with the 30 × 30 degree FAF (**F**), forming the final image (**G**). The last step of including the FAF is optional but arguably provides a better visualization of the retinal architecture, particularly in the context of inherited retinal diseases. After zooming to the highest possible magnification, the scale of 200 µm provided in the image by the Spectralis is used to create a custom ruler for each eye. In the example above, 23 pixels equals 200 microns, which was then used to measure the DFC.

We developed a method to analyze the pattern of fixation changes in patients with erratic fixation, such as patients affected by *KCNV2*-associated retinal dystrophy. This is achieved by splitting the tasks into several steps (Fig. 2), which involve defining the anatomic center of the fovea (see Figs. 2A, 2B) and finally overlaying the resulting images with the microperimetry results (Fig. 2G). The authors calculated the distance (in µm) between the anatomic foveal center and the center of the cloud of fixation points – PRLf. This methodology was used in 12 affected individuals. The PRLf is shown in Figure 1 as a cyan lozenge.

The foveal center (FC) was manually pinpointed by selecting the vertical and horizontal OCT scans that were most topographically accurate of the fovea, with its corresponding infrared image provided by the software. When present, the foveal reflex on the OCT images guided the selection of the best positioned scans. These two images were overlaid, each on a separate layer, in Adobe Photoshop (Adobe Inc; San José, CA, USA), using the blood vessels and optic nerve (ON) as landmarks, and by matching these with the aid of the inbuilt transparency function. A simplified version of the resulting image from microperimetry containing only the PRL and the IR image, and the 30-degree FAF image were then overlapped and the exact PRLf and anatomic foveal location were plotted on the FAF image. For the most accurate measurement, the scale bars provided on the FAF by the Heyex software (200 µm) were used as reference, and a custom ruler was created for each eye (which was matched to the individual pixel level). The distance between those two points (in pixels) was then measured as the shortest line between them, with the highest possible magnification, which was later converted to µm using the aforementioned custom ruler. This parameter was designated “distance from foveal center” (DFC). Figure 2 shows the process and resulting image.

**Figure 3.**
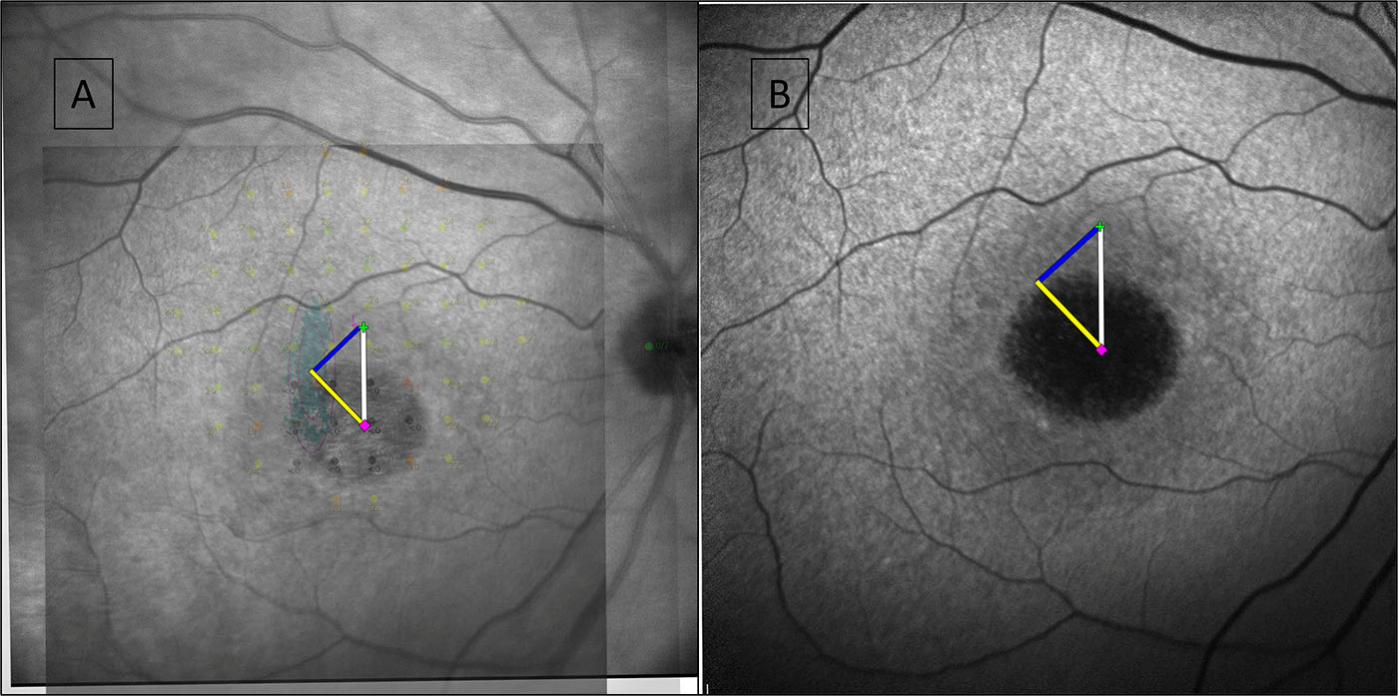
The resulting DFC image. (**A**) IR, FAF and microperimetry images overlaid in layers with opacity filters applied to demonstrate the process. (**B**) FAF image. The *white line* is the DFC at baseline, the *yellow line* is the DFC at follow-up and the *blue line* is the change of DFC during follow-up, which essentially forms a triangle.

Because the MAIA also provides the PRL_i and the PRL_f, the same image created to measure the distance between those was used, which we defined as “distance between PRL” (DPRL; µm). This essentially represents the stability of fixation. Two perpendicular lines were then intersected in the FC to define and record the position (quadrant) of the preferred fixation (Fig. 3).

Repeatability of this method was evaluated by two graders in nine randomly selected eyes. Bland-Altman and Pearson correlation coefficients were used to assess agreement.

## Results

**Figure 4.**
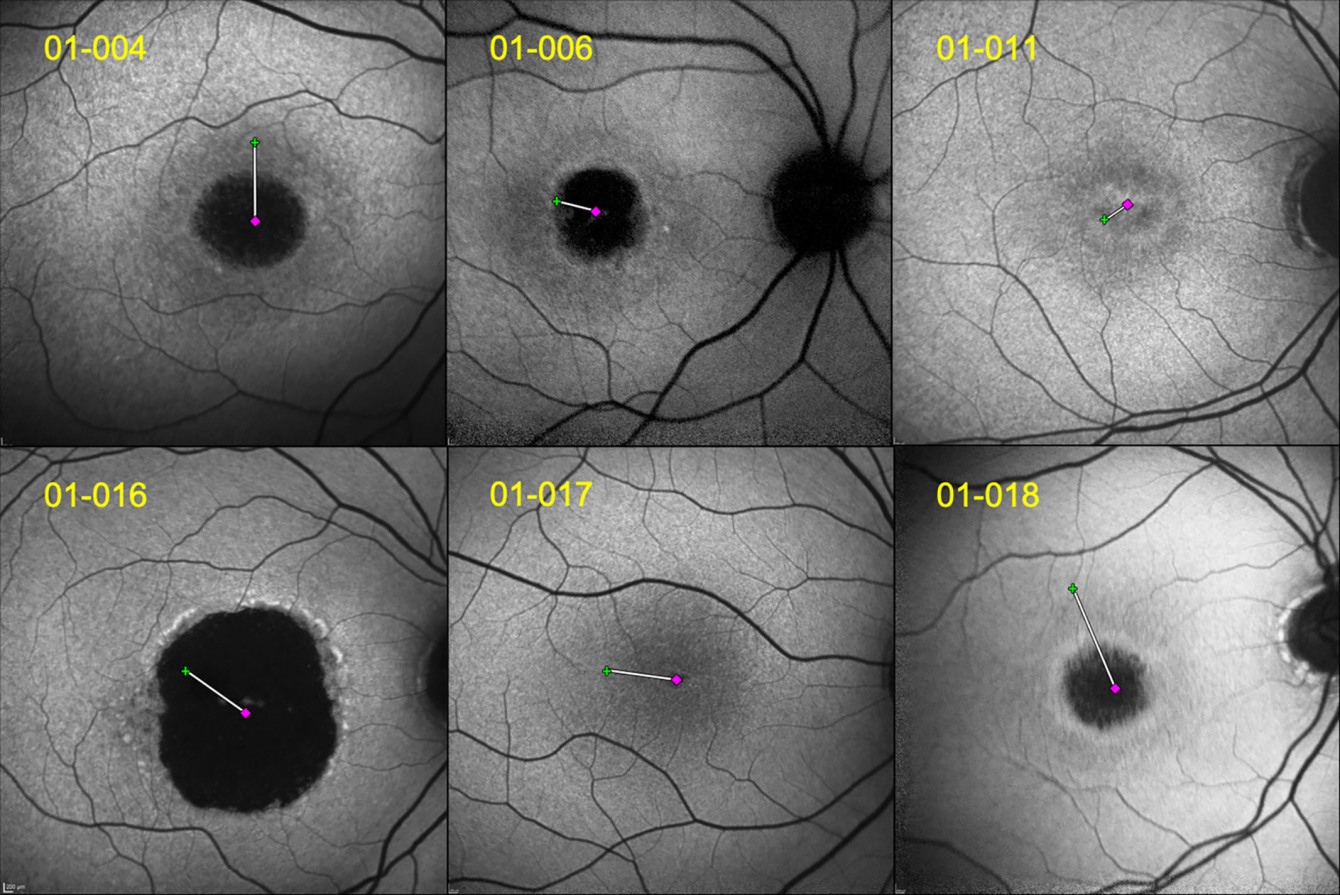
Collage demonstrating the distance of foveal center (DFC) of the right eyes of six selected patients with different stages of disease. The DFC is overlaid with a fundus autofluorescence (FAF) image, which provides a clearer view of the underlying phenotype; this is not an essential step, but it adds to the overall interpretation of the resulting images. On the *top left corner*, are the subjects’ natural history IDs. The pink lozenge refers to the foveal center (FC), the *green cross* refers to the preferred retinal locus_final (PRLf), and the *white straight line* represents the DFC.

Twelve subjects (22 eyes) completed the full study protocol. Ten subjects had bilateral testing at baseline, hence 10 matched-pairs have been used to assess interocular correlation. The mean age of the patients was 31.9 years (range = 11–54 years, SD = ±14.3). The mean BCVA (range) of all included eyes was 0.92 (range = 0.82–1.3). Figure 4 shows the results of the right eyes of six patients.

**Figure 5.**
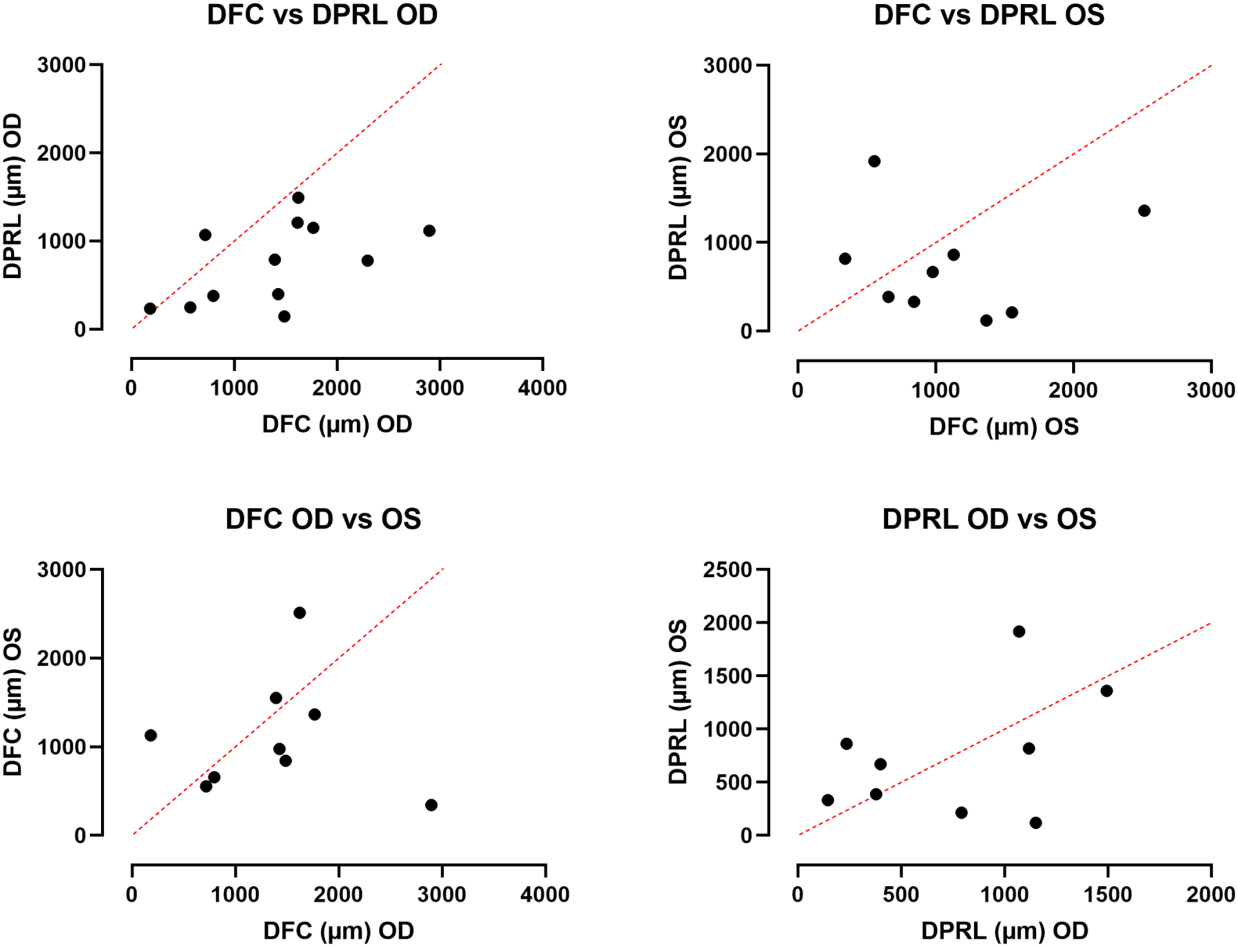
Scatter plots showing correlation between DFC versus DPRL and interocular correlation of each parameter. A line of best fit is plotted.

The mean DFC and DPRL was 1398 µm (range = 182.8–2896, SD = ±755.4) and 751.4 µm (range = 144.5–1493.3, SD = ±458.3) in the right eyes, and 1104 µm (range = 341.8–2513, SD = ±653.78) and 742.5 µm (range = 120–1918, SD = ±586.5) in the left eyes, respectively (Fig. 5). There was no significant interocular correlation (r; p; Pearson correlation coefficient) of DFC (*r* = −0.036, *P* = 0.92) or DPRL (*r* = 0.41, *P* = 0.26), or between the two variables in right (*r* = 0.519, *P* = 0.084) and left eyes (*r* = 0.014, *P* = 0.97). Similarly, there was no significant correlation (p; Pearson correlation coefficient) of age versus DFC and DPRL in the right (0.86 and 0.23, respectively) and the left eyes (0.98 and 0.59, respectively). These observations suggest that the stability of fixation (DPRL and BCEA) may not be related to the location of eccentric fixation (PRLf) and that the values of either eye are independent of the other.

Twelve eyes (57%) had PRL located in the superotemporal quadrant – with nine being the right eyes – four (19%) had inferonasal and the other four (19%) had superonasal PRLs and one (5%) eye had inferotemporal PRL.

**Figure 6.**
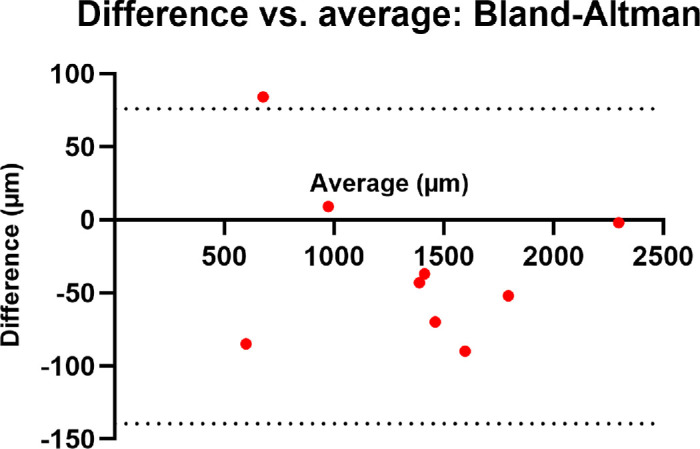
Bland-Altman analysis reveals agreement between the two graders. The *dotted lines* represent the 95% limits of agreement.

To assess the accuracy of this technique, nine eyes were then randomly selected, and the same methodology to calculate the DFC was applied cross-sectionally by a second grader (see the Table). The eyes selected were completely randomized. The mean DFC was 1339 µm (range = 555–2295, SD = ±534.5) for grader 1, and 1371 µm (range = 634.3–2297, SD = ±546.9) for grader 2, which was strongly correlated (*r* = 0.995, *P* < 0.0001; Pearson correlation coefficient). Bland-Altman reveals agreement between the 2 graders, with a bias of 31.78 µm (SD = ±55) – a difference which is not likely to be clinically relevant (Fig. 6).

**Table.** DFC Values of Each Pair of Eyes Measured by Both Graders

	DFC Measurement	
Pair #	Grader 1, µm	Grader 2, µm
1	977	968
2	1552	1642
3	555	640
4	1366	1409
5	1426	1496
6	2295	2297
7	1393	1430
8	718	634
9	1768	1820

DFC, distance from foveal center.

All measurements are in µm.

## Discussion

We present a novel method for assessing the eccentric fixation position, as applied in a cohort of patients with *KCNV2*-associated retinal dystrophy. In diseases affecting the central retina, functional deterioration may precede evidence of microstructural changes; a scenario in which further information relating to the PRL may provide further disease characterization. Similarly, being able to provide a uniform and standardized point metric – as opposed to the area of an ellipse – may increase its reliability/utility as a clinical trial outcome.

Most of the patients included herein have significant loss of outer retinal lamination in the macula, which may create difficulties to identify the most central OCT B-scan and precise location of the anatomic FC0. This could reduce the ability to accurately measure the DFC. However, despite these challenges, in the cohort herein, there was a high agreement between the two graders. Given that central retinal atrophy is characteristic in a variety of retinal diseases, such as age-related macular degeneration and Stargardt disease,^12^^–^^14^ this parameter could also be of interest in the context of these more common conditions.

Our data suggest that the PRL may not follow a specific pattern in *KCNV2*-associated retinal dystrophy. It is currently unknown if this is correlated with visual function, such as previous reports in patients with amblyopia.^5^^,^^6^ This question is the subject of ongoing work, with application to larger datasets. The majority of the right eyes had PRLs located in the superotemporal quadrant (75%), whereas the left eyes appeared to fluctuate between the infero- and superonasal quadrants (66.6%). It is unlikely that this difference could be accounted for by the cone arrangement/distribution in the human retina, as previous studies reported no significant difference in cone density between nasal and temporal meridians or between the superior and inferior meridians.^15^^,^^16^ Interestingly, the PRL location in our cohort is in keeping with a study published in 2018 by Chiang et al. that analyzed the fixation behavior in a range of macular dystrophies.^17^ The authors also reported that PRLs were more common in the superior retinal quadrant.

Finally, the specific sample that the authors have selected in the present work is likely optimal to assess the relevancy of this methodology. It consists of patients with a specific form of inherited retinal disease that has been well described and is highly symmetric both from a functional and structural perspective.^18^^,^^19^ This disease is characterized by a universally decreased visual acuity and central ellipsoid zone disruption. Thus, it is likely that the subjects used a different retinal locus during the visual acuity test, hampering our ability to compare the relationship between retinal eccentric and fixation variability.

## Conclusions

In the present study, the tests were standardized following well described protocols and acquired prospectively by an expert technical team. Similarly, these data suggest that these are reliable parameters, which could be of relevance in the context of clinical trials. However, the sample size is small and only to be taken as an example of this methods’ application, with a larger and more heterogenous dataset being necessary to extrapolate any conclusions regarding the location of the PRL in patients with central retinal disease. Its correlation with other functional and structural outcomes remains to be explored. Despite these limitations, these findings provide a framework for further development.

## References

[1] Csaky KG, Richman EA, Ferris FL 3rd. Report from the NEI/FDA Ophthalmic Clinical Trial Design and Endpoints Symposium. *Invest Ophthalmol Vis Sci*. 2008; 49(2): 479–489. 18234989 10.1167/iovs.07-1132

[2] de Guimaraes TAC, de Guimaraes IMC, Ali N, et al. In-depth retinal sensitivity assessment with the MP3 type S microperimeter: a methods study. *Transl Vis Sci Technol*. 2024; 13(4): 14. 10.1167/tvst.13.4.14 PMC11008759 38591946

[3] Whittaker SG, Budd J, Cummings R. Eccentric fixation with macular scotoma. *Invest Ophthalmol Vis Sci*. 1988; 29(2): 268–278. 3338884

[4] Schuchard RA, Naseer S, de Castro K. Characteristics of AMD patients with low vision receiving visual rehabilitation. *J Rehabil Res Dev*. 1999; 36(4): 294–302. 10678452

[5] García-García M, Belda JI, Schargel K, et al. Optical coherence tomography in children with microtropia. *J Pediatr Ophthalmol Strabismus*. 2018; 55(3): 171–177. 29384563 10.3928/01913913-20171026-01

[6] Jin J, Apple A, Friess A, et al. Using OCT fixation shift to assess eccentric fixation in children with residual amblyopia. *Transl Vis Sci Technol*. 2020; 9(12): 30. 10.1167/tvst.9.12.30 PMC7691785 33262904

[7] Greenstein VC, Santos RA, Tsang SH, et al. Preferred retinal locus in macular disease: characteristics and clinical implications. *Retina*. 2008; 28(9): 1234–1240. 18628727 10.1097/IAE.0b013e31817c1b47 PMC2749558

[8] Hatef E, Hanout M, Moradi A, et al. Longitudinal comparison of visual acuity as measured by the ETDRS chart and by the potential acuity meter in eyes with macular edema, and its relationship with retinal thickness and sensitivity. *Eye (Lond)*. 2014; 28(10): 1239–1245. 25104744 10.1038/eye.2014.182 PMC4194343

[9] Carpineto P, Ciancaglini M, Di Antonio L, Gavalas C, Mastropasqua L. Fundus microperimetry patterns of fixation in type 2 diabetic patients with diffuse macular edema. *Retina*. 2007; 27(1): 21–29. 17218911 10.1097/01.iae.0000256658.71864.ca

[10] Bonnabel A, Bron AM, Isaico R, Dugas B, Nicot F, Creuzot-Garcher C. Long-term anatomical and functional outcomes of idiopathic macular hole surgery. The yield of spectral-domain OCT combined with microperimetry. *Graefes Arch Clin Exp Ophthalmol*. 2013; 251(11): 2505–2511. 23620091 10.1007/s00417-013-2339-y

[11] de Guimaraes TAC, de Guimaraes IMC, Muthiah MN, Kalitzeos A, Michaelides M. Retinal sensitivity in KCNV2-associated retinopathy. *Invest Ophthalmol Vis Sci*. 2025; 66(1): 26. 10.1167/iovs.66.1.26 PMC11730890 39792073

[12] Strauss RW, Kong X, Ho A, et al. Progression of Stargardt disease as determined by fundus autofluorescence over a 12-month period: ProgStar Report No. 11. *JAMA Ophthalmol*. 2019; 137(10): 1134–1145. 31369039 10.1001/jamaophthalmol.2019.2885 PMC6681653

[13] Schmitz-Valckenberg S, Brinkmann CK, Alten F, et al. Semiautomated image processing method for identification and quantification of geographic atrophy in age-related macular degeneration. *Invest Ophthalmol Vis Sci*. 2011; 52(10): 7640–7646. 21873669 10.1167/iovs.11-7457

[14] Schmitz-Valckenberg S, Sahel JA, Danis R, et al. Natural history of geographic atrophy progression secondary to age-related macular degeneration (Geographic Atrophy Progression Study). *Ophthalmology*. 2016; 123(2): 361–368. 26545317 10.1016/j.ophtha.2015.09.036

[15] Song H, Chui TYP, Zhong Z, Elsner AE, Burns SA. Variation of cone photoreceptor packing density with retinal eccentricity and age. *Invest Ophthalmol Vis Sci*. 2011; 52(10): 7376–7384. 21724911 10.1167/iovs.11-7199 PMC3183974

[16] Legras R, Gaudric A, Woog K. Distribution of cone density, spacing and arrangement in adult healthy retinas with adaptive optics flood illumination. *PLoS One*. 2018; 13(1): e0191141. 29338027 10.1371/journal.pone.0191141 PMC5770065

[17] Chiang W-Y, Lee J-J, Chen Y-H, et al. Fixation behavior in macular dystrophy assessed by microperimetry. *Graefes Arch Clin Exp Ophthalmol*. 2018; 256(8): 1403–1410. 29948177 10.1007/s00417-018-4006-9 PMC6060756

[18] Georgiou M, Fujinami K, Vincent A, et al. KCNV2-associated retinopathy: detailed retinal phenotype and structural endpoints-KCNV2 Study Group Report 2. *Am J Ophthalmol*. 2021; 230: 1–11. 33737031 10.1016/j.ajo.2021.03.004 PMC8710866

[19] Georgiou M, Robson AG, Fujinami K, et al. KCNV2-associated retinopathy: genetics, electrophysiology, and clinical course-KCNV2 Study Group Report 1. *Am J Ophthalmol*. 2021; 225: 95–107. 33309813 10.1016/j.ajo.2020.11.022 PMC8186730

